# Progenitor cells, microglia, and non-coding RNAs: Orchestrators of glioblastoma pathogenesis and therapeutic resistance

**DOI:** 10.1016/j.ncrna.2025.07.007

**Published:** 2025-08-05

**Authors:** Adil Husain, Firoz Ahmad, Sandeep Pandey, Tarun Kumar Upadhyay, Sojin Kang, Min Choi, Jinwon Choi, Moon Nyeo Park, Bonglee Kim

**Affiliations:** a Department of Biochemistry, Babu Banarasi Das College of Dental Sciences, Babu Banarasi Das University, Lucknow, 226028, Uttar Pradesh, India; b Department of Physiological Sciences, Oklahoma State University, Oklahoma City, Stillwater, USA; c Department of Biochemistry, King George's Medical University, Lucknow, 226003, Uttar Pradesh, India; d Department of Life Sciences, Parul Institute of Applied Sciences & Research and Development Cell, Parul University, Vadodara, 391760, Gujarat, India; e Department of Pathology, College of Korean Medicine, Kyung Hee University, Hoegidong, Dongdaemungu, Seoul, 05253, Republic of Korea

**Keywords:** Microglia, Non-coding RNA, Glioblastoma, Progenitor cells

## Abstract

Glioblastoma (GB) remains a major challenge owing to its extremely aggressive nature and resistance to conventional therapies. This review focuses on the intricate roles of progenitor cells, microglia, and non-coding RNAs (ncRNAs) in orchestrating GB pathogenesis and therapy resistance. Glioma stem cells (GSCs), derived from progenitor cells, are important drivers of tumor initiation and recurrence and exhibit remarkable plasticity and resistance to treatment. Microglia, the immune cells of the brain, are hijacked by GB cells to create an immunosuppressive microenvironment that supports tumor growth and resistance to therapy. Non-coding RNAs, including microRNAs and long noncoding RNAs, regulate multiple resistance mechanisms by modulating gene expression and influencing the interactions between progenitor cells and microglia. This review highlights new insights into these interconnected signaling pathways and explores potential therapeutic strategies targeting these molecular players to overcome treatment resistance and improve outcomes in patients with GB.

## 1 Introduction

Glioblastoma (GB) is the most aggressive and deadly form of primary brain tumor in adults, accounting for over 50 % of all gliomas [1]. It is characterized by rapid growth, strong invasion of the surrounding brain tissue, and marked cellular heterogeneity, making treatment difficult [2]. Current therapeutic approaches include maximal surgical resection followed by concurrent chemoradiotherapy and chemotherapy, usually with temozolomide (TMZ) [3,4]. However, even with aggressive treatment, the prognosis remains dismal, with a median survival of 12–15 months and a 5-year survival rate of less than 10 % [1]. A major challenge is the incomplete surgical removal of the tumor due to its diffuse infiltration into healthy brain tissue, as well as the development of resistance to radiation and chemotherapy [5]. Furthermore, the tumor microenvironment (TME) and molecular complexity, including genetic and epigenetic alterations, contribute to poor treatment outcomes. Understanding the intricate cellular and molecular mechanisms that drive GB pathogenesis and resistance to therapy is crucial for developing new and effective treatment strategies.

This review provides a comprehensive overview of three key components of GB progression: progenitor cells, microglia, and non-coding RNAs (ncRNAs). Progenitor cells, particularly glioma stem-like cells (GSCs), are a source of tumor recurrence and resistance because of their ability to self-renew and differentiate into various cell types within the tumor. Microglia, the resident immune cells of the brain, are often reprogrammed by the tumor to create an immunosuppressive environment, aiding in invasiveness and resistance to therapies. In addition, ncRNAs, such as microRNAs (miRNAs) and long non-coding RNAs (lncRNAs), have emerged as crucial regulators of gene expression, modulating pathways that drive GB malignancy and resistance to conventional treatments.

By focusing on these components, we discuss how their interactions contribute to the development and persistence of GB and how they can be targeted for novel therapeutic interventions. The overarching goal is to highlight promising research avenues that could lead to more effective targeted therapies, improve patient outcomes, and overcome current therapeutic challenges.

## 2 Progenitor cells in GB: drivers of tumorigenesis and resistance

### 2.1 Progenitor cells in the Central Nervous System (CNS)

**Fig. 1:**
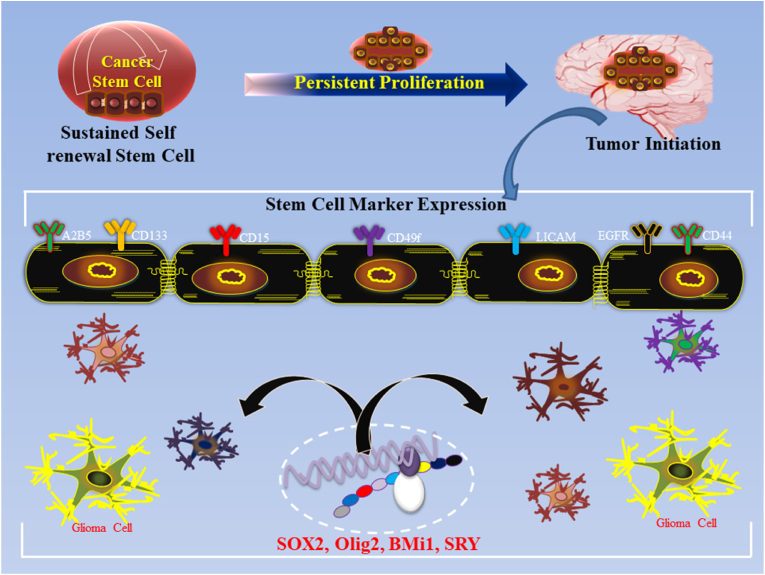
CSC functional characteristics include sustained self-renewal, persistent proliferation, and tumor initiation upon intracranial transplantation, defining their role in GB. Additionally, CSCs share similarities with somatic stem cells, exhibiting tissue/tumor-specific frequency, stem cell marker expression (e.g., Bmi1, Olig2, Sox2), and multilineage progeny potential.

Neural progenitor cells (NPCs) are essential components of the CNS and are responsible for generating various types of neural cells during brain development. They exhibit the capacity for self-renewal and differentiation, and play a critical role in maintaining brain homeostasis and responding to injury [6,7]. However, in the GB, certain progenitor cells, particularly GSCs, undergo an aberrant transformation, becoming key drivers of tumorigenesis (Fig. 1). GSCs share many characteristics with normal NPCs, including self-renewal and multipotency, but they also exhibit enhanced survival, proliferation, and resistance to standard therapies [7]. This malignant stem-like population is thought to be responsible for the initiation, growth, and recurrence of GB, making them pivotal players in the aggressive nature [7].

**Fig. 2:**
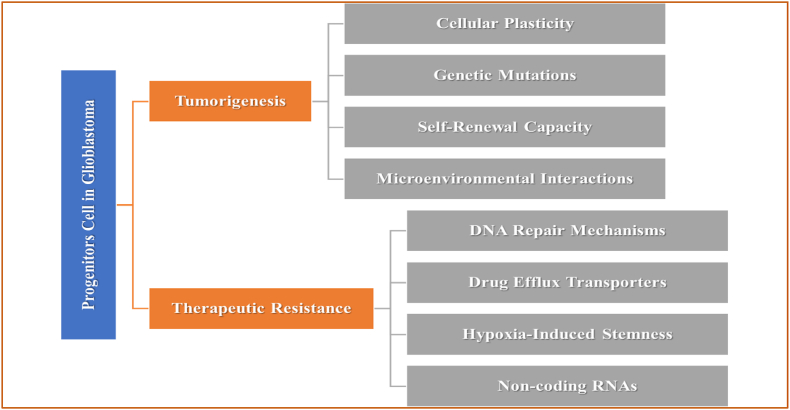
Role of Progenitor Cells in GB. Cellular plasticity, genetic mutations, self-renewal capacity, and interactions with the microenvironment are key drivers of tumorigenesis, while DNA repair mechanisms, drug efflux transporters, and hypoxia-induced stemness and regulatory non-coding RNAs contribute to therapeutic resistance in GB. Progenitor cells play a dual role in GB by promoting aggressiveness and survival challenges.

### 2.2 Transformation of progenitor cells into GSCs

The transformation of progenitor cells into GSCs is a complex process driven by both genetic and epigenetic alterations [8]. Mutations in key oncogenes and tumor suppressor genes such as *TP53* and *IDH1* are common in gliomas and contribute to the malignant transformation of these cells [9]. *TP53* mutations, a hallmark of many cancers, disrupt normal cell cycle regulation and promote genomic instability. Meanwhile, mutations in *IDH1*, particularly in lower-grade astrocytomas and oligodendrogliomas, are linked to the production of the oncometabolite 2-hydroxyglutarate, which interferes with cellular differentiation and promotes a stem-like state [[10], [11], [12]]. Epigenetic modifications such as DNA methylation and histone modification further contribute to the stem-like phenotype of GB cells. These changes alter gene expression patterns and maintain progenitor cells in an undifferentiated and proliferative state [13]. In addition, the TME plays a crucial role in the transformation and maintenance of GSCs. Hypoxia is a hallmark of most malignancies, including GB-TME, and has been connected to worse patient outcomes and aggressive metastatic features. GSC survival and stemness are supported by the hypoxic condition they occasionally encounter, which is thought to be regulated by hypoxia-inducible factor signaling [14]. Cytokines and growth factors within the TME, including interleukin-6 (IL-6) and transforming growth factor-beta (TGF-β), further promote the plasticity and self-renewal capabilities of progenitor cells, enabling their adaptation to the tumor niche [[15], [16], [17]].

### 2.3 Progenitor cells and tumor growth

GSCs are pivotal not only in tumor initiation but also in driving the continuous growth and invasion of GB [18]. These cells display enhanced proliferative potential and are highly invasive, contributing to the diffuse infiltration of the GB throughout the brain [19,20]. Unlike more differentiated tumor cells, GSCs possess the ability to migrate along white matter tracts and blood vessels, enabling them to evade surgical resection and seed new tumor foci [21]. The presence of GSCs within the tumor mass also contributes to its heterogeneity, which is a defining feature of GB. GSCs can differentiate into various cell types within the tumor, leading to a heterogeneous population of cells with varying levels of susceptibility to treatment [2,22]. This heterogeneity is a significant factor in the development of therapy resistance, as GSCs can survive treatments that effectively eliminate differentiated tumor cells. As a result, GSCs are often implicated in tumor recurrence as they can repopulate the tumor following therapy [5,23].

### 2.4 Progenitor cells and therapy resistance

Progenitor cells, particularly GSCs, are highly resistant to conventional therapies, including radiation and chemotherapy. This resistance is attributed to several factors, including enhanced DNA repair mechanisms, slow cell cycle progression, and activation of survival pathways that protect cells from apoptosis [[24], [25], [26]]. For instance, GSCs exhibit increased expression of DNA repair proteins such as MGMT, which confers resistance to TMZ, the standard chemotherapeutic agent used in GB treatment [27,28]. Furthermore, GSCs often reside in protective niches within tumors, such as perivascular or hypoxic regions, where they are shielded from therapeutic agents and radiation [5,[24], [25], [26]].

The cellular plasticity of GSCs also plays a significant role in resistance to therapy. These cells can dynamically switch between stem-like and differentiated states in response to therapeutic pressure, allowing them to survive treatment and re-establish the tumor. This adaptability makes targeting GSCs a crucial focus for the development of new therapeutic strategies [22,29,30].

Emerging therapies targeting key signaling pathways involved in GSC maintenance and self-renewal, such as the Notch, Wnt, and Sonic Hedgehog pathways, have shown promise in preclinical studies [26,31]. These pathways are critical for the regulation of stemness and differentiation of both normal progenitor cells and GSCs. Inhibitors of these pathways, such as gamma-secretase inhibitors (targeting Notch signaling), are being investigated as potential therapies to specifically target the GSC population, thereby overcoming resistance and reducing the likelihood of tumor recurrence [26,[31], [32], [33]].

## 3 Microglia in GB: tumor-associated immune cells

### 3.1 Microglia and their role in brain homeostasis

Microglial brain resident immune cells are pivotal in maintaining the health and homeostasis of the CNS. Originating from yolk sac progenitors, these unique cells constitute approximately 10–15 % of the total cells in the brain. Unlike other immune cells that circulate in the bloodstream, microglia are strategically positioned throughout the CNS, enabling them to respond quickly to various stimuli, including injury, infection, and disease [34,35]. In their resting state, microglia exhibit a characteristic ramified morphology with long, thin processes that extend into the surrounding environment. This morphology allows them to constantly survey the CNS for changes or damage. Through their highly motile processes, microglia engage in the active surveillance of synapses, contributing to synaptic pruning, a critical process during development that eliminates excess synapses to optimize neural circuit functions [35,36].

In addition to synaptic pruning, microglia play an essential role in neurogenesis, supporting the survival and maturation of new neurons. When faced with injury or pathological changes, the microglia undergo rapid activation. This activation leads to a transformation from a resting ramified state to an amoeboid shape, which enhances their ability to engulf cellular debris, dead cells, and pathogens. Activated microglia release a variety of pro-inflammatory cytokines, including tumor necrosis factor-alpha (TNF-α), interleukin-1 beta (IL-1β), and IL-6. These cytokines play essential roles in recruiting other immune cells to the injury site, thereby initiating the healing process [35,37]. While acute microglial activation is crucial for repair and recovery, prolonged or dysregulated activation can lead to chronic neuroinflammation, which is associated with various neurodegenerative diseases, such as Alzheimer's disease and multiple sclerosis. Thus, maintaining a balance in microglial activation is critical for brain health, underscoring their dual roles as protectors and potential contributors to pathology [36,37].

### 3.2 Microglial infiltration into the GB microenvironment

In the context of GB, which is one of the most aggressive forms of brain cancer, the role of microglia has become increasingly complex. GB is characterized by its highly infiltrative nature, extensive cellular heterogeneity, and unique TME that includes not only tumor cells, but also stromal cells, vascular components, and immune cells, particularly microglia. Microglial infiltration into the GB microenvironment is a dynamic process influenced by multiple factors. GB cells release a plethora of signaling molecules, including cytokines, chemokines, and extracellular vesicles, which play critical roles in recruiting and reprogramming microglia [[37], [38], [39]]. For instance, the secretion of TGF-β by GB cells is a key factor that drives microglial activation and polarization toward a tumor-promoting phenotype. Once recruited, microglia undergo significant reprogramming, altering their function and phenotype in response to TME. This reprogramming often results in a shift from a protective role to pro-tumor activities [35,36]. Factors such as interleukin-10 (IL-10), a cytokine with anti-inflammatory properties, and the release of extracellular vesicles carrying miRNAs and other bioactive molecules from tumor cells can further influence microglial behavior. This interaction results in a population of tumor-associated microglia that is markedly different from their resting counterparts. These tumor-associated microglia often exhibit features characteristic of the M2-phenotype, which is associated with immune suppression, tissue repair, and promotion of tumor growth [36,37]. In this altered state, microglia can support GB progression by enhancing the survival and proliferation of tumor cells, promoting angiogenesis, and facilitating the invasion of the surrounding brain tissue. Moreover, the TME can create a feedback loop that perpetuates microglial activation. As microglia become more involved in supporting tumor growth, they may release additional signals that further enhance GB cell proliferation and survival, creating a vicious cycle that complicates treatment efforts [39,40].

### 3.3 Microglial polarization and GB progression

Microglia are well-known for their remarkable plasticity, which enables them to adopt various functional states in response to environmental cues. In the context of GB, microglial polarization can be broadly classified into two main phenotypes, M1 and M2. The balance between these two phenotypes plays a critical role in determining the overall outcome of tumor-host interaction [37,40].

**M1 Microglia: Pro-Inflammatory Phenotype:** M1 microglia are classically activated in response to pro-inflammatory signals and are associated with antitumor immune responses. They secrete a variety of pro-inflammatory cytokines such as IL-12 and interferon-gamma (IFN-γ), which can enhance the activity of other immune cells, including T-cells and natural killer (NK) cells. This pro-inflammatory environment can inhibit tumor growth and promote tumor cell apoptosis. However, in GB, the M1 response is often overshadowed by the predominance of M2 microglia, which limits the effectiveness of this anti-tumor response. The transition from M1 to M2 is facilitated by the TME, which is rich in immunosuppressive factors [36,41].

**M2 Microglia: Tumor-Promoting Phenotype:** M2 microglia, on the other hand, are associated with tissue repair and resolution of inflammation. They produce anti-inflammatory cytokines, such as IL-10 and TGF-β, which can suppress the activity of effector immune cells and promote tumor survival. In the GB microenvironment, M2 microglia contribute to several tumor-promoting functions. For example, M2 microglia secrete various angiogenic factors, such as vascular endothelial growth factor (VEGF), which promotes the formation of new blood vessels [41]. This is crucial for tumor growth as it ensures that GB cells receive the necessary nutrients and oxygen to thrive. Immunosuppression: By producing anti-inflammatory cytokines, M2 microglia creates a microenvironment that inhibits effective antitumor immune responses [[40], [41], [42]]. This immunosuppression can lead to evasion of immune surveillance, allowing GB cells to proliferate and metastasize more easily. Tumor Invasion: M2 microglia can facilitate the invasion of GB cells into the surrounding brain tissue by remodeling the extracellular matrix [42,43]. This remodeling process involves the secretion of matrix metalloproteinases (MMPs), which degrade the components of the extracellular matrix, thereby enabling tumor cells to migrate more freely. The polarization of microglia toward the M2 phenotype is thus a key factor in GB progression [44]. This shift not only promotes tumor growth but also complicates treatment approaches, as targeting the immune response becomes increasingly challenging in a microenvironment that favors tumor survival.

### 3.4 Microglia and therapeutic resistance

The interplay between GB cells and microglia significantly contributes to therapeutic resistance, which is a major challenge in the treatment of aggressive cancer. GBs are notoriously resistant to conventional therapies, including surgery, chemotherapy, and radiation, and microglia play several roles in this resistance.

**Mechanisms of Immune Evasion:** One of the primary mechanisms by which microglia contribute to immune evasion is the secretion of growth factors that enhance GB cell survival. For example, insulin-like growth factor-1 is a potent survival factor that can be released by microglia, promoting resistance to apoptosis in GB cells even when subjected to chemotherapeutic agents [44,45]. This interaction effectively enables tumor cells to withstand treatments that would typically induce cell death. Additionally, microglia can upregulate immune checkpoint proteins such as programmed death-ligand 1 (PD-L1), which inhibits T-cell activation and promotes an immunosuppressive environment. By expressing PD-L1, microglia can contribute to the evasion of immune surveillance, allowing GB cells to proliferate unchecked [46,47].

**Radiation Resistance:** Microglia have also been implicated in radiation resistance, which is a significant concern for GB therapy. Following radiation treatment, activated microglia secrete neuroprotective factors and cytokines that aid tumor cell survival. For instance, microglial release of IL-6 can activate signaling pathways in GB cells that promote survival and proliferation, thereby counteracting the intended effects of radiation therapy. This radiation-induced activation of microglia can lead to a vicious cycle, wherein tumor cells stimulate microglial activation and, in turn, activated microglia support the survival of tumor cells. This cycle not only undermines the efficacy of radiation therapy but also creates a challenging environment for the development of novel treatment strategies [5,23,46].

**Potential Therapeutic Strategies Targeting Microglia:** Given the significant role of microglia in GB progression and therapeutic resistance, targeting these cells is a promising avenue for improving treatment outcomes. Several strategies have been proposed:

***CSF-1R Inhibitors:*** Colony-stimulating factor-1 receptor (CSF-1R) inhibitors aim to disrupt the recruitment and activation of microglia in the TME. By inhibiting CSF-1R, these agents can reduce the population of pro-tumor M2 microglia, potentially restoring a more protective immune environment [48].

***Immune Checkpoint Blockade:*** Combining immune checkpoint inhibitors with strategies to modulate microglial behavior may enhance anti-tumor immunity. For example, by blocking PD-L1 interactions, immune checkpoint blockade can reinvigorate T-cell responses, potentially overcoming the immunosuppressive effects of tumor-associated microglia [49,50].

***Reprogramming Microglia******:*** Approaches aimed at reprogramming microglia from the M2 to the M1 phenotype hold promise for enhancing antitumor responses. Therapeutic agents that promote M1 polarization or inhibit M2 signaling pathways could shift the balance toward a more favorable immune environment for combating GB [51]. In GB, a variety of therapeutic agents have demonstrated the ability to rewire tumor-associated microglia (TAMs) from the M2 to the M1 phenotype. CSF-1R blockers (e.g., PLX3397) inhibit the survival of M2 macrophages, thereby diminishing their immunosuppressive impact. [52]. STAT3 inhibitors WP1066, STAT3 inhibitors, block M2-polarizing transcriptional programs and promote M1 gene expression [53]. By boosting the release of pro-inflammatory cytokines, TLR agonists stimulate innate immune signaling. PI3Kγ modulators (e.g., IPI-549) interfere with immunosuppressive signaling in myeloid cells, reprogramming TAMs [54].

***Combination therapies******:*** Combination therapies that incorporate standard treatments (such as chemotherapy and radiation) with microglial-targeted therapies could provide synergistic effects, improve treatment efficacy, and overcome resistance mechanisms [55,56].

## 4 NcRNAs in GB: key regulators of pathogenesis and resistance

NcRNAs are a vital component of the genome, encompassing a wide array of RNA molecules that do not translate into proteins but play crucial regulatory roles in gene expression and cellular functions. Their importance in various biological processes, particularly cancer, has garnered significant attention in recent years. Below, we explore the types of ncRNAs, their mechanisms of action, and their implications in diseases, such as GB, cancer, and TB [[57], [58], [59], [60]].

### 4.1 MiRNAs and their role in GB

They are short, typically 20–22 nucleotides in length, single-stranded RNA molecules that primarily function in post-transcriptional regulation. MiRNAs bind to complementary sequences in target mRNAs, leading to mRNA degradation or translational repression. This process modulates gene expression and can have profound effects on various cellular functions, including proliferation, differentiation, and apoptosis. MiRNAs are emerging as vital regulators in GB, influencing many aspects of tumor biology [57,61,62]. Research has identified several key miRNAs that are significantly implicated in GB pathogenesis.

**miR-21:** Often referred to as an “oncomiR”, miR-21 is frequently overexpressed in GB tissues and is associated with aggressive tumor behavior [63]. It promotes cell proliferation and invasion by targeting tumor suppressor genes, such as phosphatase and tensin homolog (PTEN), and RECK (reversion-inducing cysteine-rich protein with Kazal motifs). Upregulation of miR-21 correlates with poorer patient prognosis, highlighting its potential as a therapeutic target.

**miR-10b:** This miRNA enhances the invasive properties of GB cells. By downregulating HOXD10, a gene known for its tumor-suppressive functions, miR-10b facilitates tumor cell migration and invasion, thereby contributing to the aggressive nature of GB [64].

**miR-34a:** Acting as a tumor suppressor, miR-34a regulates critical pathways involved in cell cycle control and apoptosis. Its expression is frequently downregulated in GB, leading to unchecked cell proliferation and enhanced survival of tumor cells in response to stress [65,66].

**miR-181a**: In GB, miR-181a is a brain-enriched miRNA that has two roles. Most of the research focuses on its tumor-suppressive properties. GB tissues and GSCs commonly downregulate it; restoring it inhibits tumor cell invasion and proliferation and encourages apoptosis. One of the main ways that miR-181a works is by specifically downregulating BCL-2, a crucial anti-apoptotic protein, which makes GB cells more susceptible to TMZ-induced apoptosis [67]. Additionally, miR-181a inhibits inflammation and slows the growth of tumors by targeting genes involved in the NF-κB signaling pathway. Additionally, by preventing DNA damage repair mechanisms, miR-181a may improve the response of GB cells to radiation therapy, according to some research [68].

The mechanisms by which miRNAs exert their effects on GB include the following.

***Regulation of Proliferation:*** MiRNAs, such as miR-34a, target genes, are involved in cell cycle progression. By inhibiting these targets, miR-34a can prevent tumor cells from progressing through the cell cycle, thereby reducing their proliferation. Conversely, loss of miR-34a expression can lead to enhanced cell growth.

***Promotion of Invasion:*** MiRNAs such as miR-10b facilitate GB invasion by targeting cell adhesion molecules and extracellular matrix components [65,66]. This regulation allows tumor cells to detach from their primary site and invade the surrounding tissues, a hallmark of GB aggressiveness.

***Maintenance of Stemness:*** MiRNAs are crucial for the maintenance of cancer stem cell characteristics that are linked to tumor recurrence and treatment resistance. For example, miR-21 promotes stemness in GB cells, enabling them to survive in harsh microenvironments and resist therapy [65,69].

MiRNAs also play significant roles in GB resistance to therapies:

1. ***Drug Efflux Mechanisms:*** miRNAs influence the expression of ATP-binding cassette (ABC) transporters, which are responsible for drug efflux. The overexpression of specific miRNAs can enhance the expression of these transporters, leading to decreased intracellular concentrations of chemotherapeutic agents and reduced drug efficacy.
2. ***Evasion of Apoptosis:*** By down-regulating pro-apoptotic factors and up-regulating anti-apoptotic factors, miRNAs enable GB cells to evade programmed cell death. This mechanism is particularly important in the context of chemotherapy and radiation, where the induction of apoptosis is a primary therapeutic goal [65,70] (see Fig. 2).

### 4.2 LncRNAs and their role in GB

**Fig. 3:**
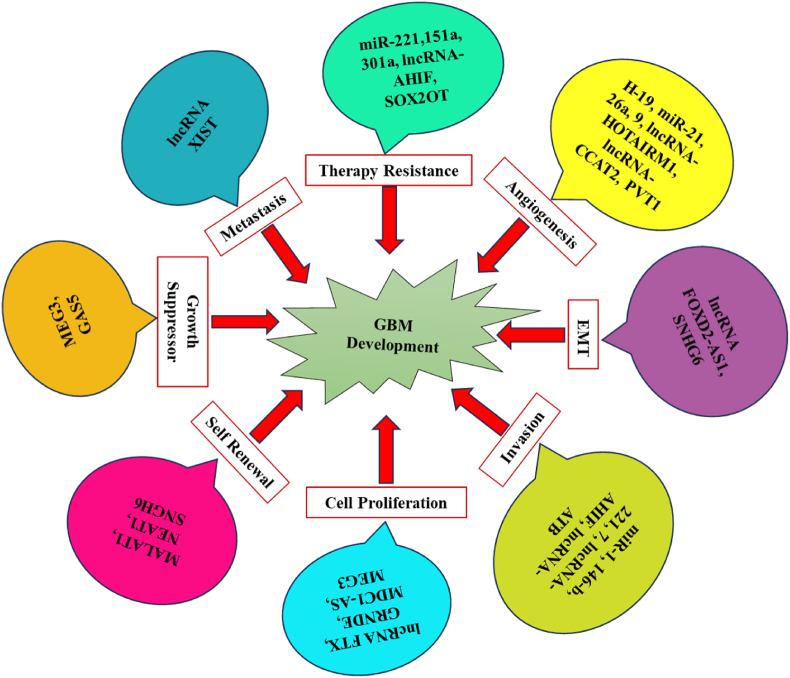
Schematic summary of various lncRNAs and miRNAs that play important roles in regulating glioma Angiogenesis, EMT, Invasion, Cell Proliferation, self-renewal, growth suppressor, metastasis, and therapy resistance.

Defined as ncRNAs longer than 200 nucleotides, lncRNAs exhibit a wide range of biological activities. They can interact with chromatin, transcription factors, and other RNA molecules, influencing gene expression at multiple levels. LncRNAs are involved in regulating cellular processes such as cell cycle progression, differentiation, and responses to stress [[58], [59], [60],^71^]. LncRNAs are increasingly recognized for their roles in GB pathogenesis. Key lncRNAs involved in GB are summarized in Fig. 3, and some of them are discussed below.

***HOTAIR:*** Homeobox transcript antisense intergenic RNA (HOTAIR) is a well-studied lncRNA associated with poor prognosis in GB [72,73]. HOTAIR facilitates tumor metastasis through chromatin remodeling, which alters the expression of genes involved in invasion and migration. Its overexpression correlates with increased tumor aggressiveness and enhanced metastatic potential [74]. The HOTAIR serves as an epigenetic scaffold and is upregulated in GB. Its 5′ domain binds the LSD1/CoREST complex and Polycomb repressive complex 2 (PRC2; EZH2/SUZ12/EED) to cause H3K4 demethylation and H3K27 trimethylation at target loci [75]. This silences genes (e.g., at the HOXD locus), promoting tumor progression. In resistant GB cells, HOTAIR also acts as a competing endogenous RNA: it sponges miR-214, leading to activation of Wnt/β-catenin signaling and upregulation of MGMT. The net effect is enhanced DNA repair and TMZ resistance via a miR-214/β-catenin/MGMT axis [76,77].

***MALAT1:*** Metastasis-associated lung adenocarcinoma transcript 1 (MALAT1) is overexpressed in GB and plays a critical role in regulating cell proliferation and migration, and correlates with poor outcome. MALAT1 promotes TMZ chemoresistance by acting as a molecular “sponge” for tumor-suppressive miRNAs, thereby modulating the expression of target genes that control cell cycle progression and tumor growth [78]. MALAT1 binds and downregulates miR-203, a miRNA that normally targets thymidylate synthase (TS) mRNA. By repressing miR-203, MALAT1 derepresses TS expression, enabling enhanced DNA synthesis and survival under TMZ. Consequently, MALAT1 knockdown restores miR-203 levels, reduces TS, and resensitizes GB cells to TMZ [79].

***NEAT1:*** Nuclear paraspeckle assembly transcript 1 (NEAT1) is essential for the formation of paraspeckles, nuclear structures involved in gene expression regulation. NEAT1 contributes to GB progression by promoting cell survival, influencing the stress response, and modulating immune responses within the TME [80,81]. NEAT1 is highly regulated in GB, and its expression is correlated with poor prognosis and increased tumour aggressiveness. NEAT1 promotes survival of tumour cells by modulating stress response pathways, such as those activated by hypoxia and DNA damage, allowing the GB cells to withstand the harsh microenvironment and the therapeutic insults of chemotherapy [82].

Mechanistically, NEAT1 acts as a molecular reservoir for several tumor suppressor miRNAs such as miR-449b-5p, which de-activate key oncogenic targets such as c-Met and STAT3, which are key players in cell proliferation, invasion, and apoptosis [83]. NEAT1 also modulates the immune landscape in the TME by modulating inflammatory mediators and promoting immunosuppression, which is conducive to tumour progression. In addition, NEAT1 has been shown to confer resistance to TMZ by increasing the ability to repair DNA and maintaining the characteristics of stem cells by interacting with the EZH2-PRC2 complex, resulting in epigenetic silencing of pro-apoptotic genes [84].

***SBF2-AS1:*** The antisense lncRNA SBF2-AS1 is highly expressed in TMZ-resistant GB and is secreted in exosomes. Its transcription is driven by ZEB1, which binds the SBF2-AS1 promoter. Overexpression of SBF2-AS1 in GB cells increases TMZ resistance, whereas knockdown sensitizes cells [85]. Mechanistically, SBF2-AS1 functions as a competitive endogenous RNA for miR-151a-3p: by sponging miR-151a-3p, it relieves repression of XRCC4, a DNA double-strand break repair protein. The elevated XRCC4 enhances DNA repair capacity and survival after TMZ-induced damage. Exosomal SBF2-AS1 from resistant tumors can transfer this resistance to other GB cells, and high serum levels predict poor TMZ response [85,86].

***Lnc-TALC:*** The lncRNA lnc-TALC (“TMZ-associated lncRNA in glioblastoma recurrence”) is upregulated in TMZ-resistant GB cells. It acts as a sponge for miR-20b-3p, thereby de-repressing c-Met (MET) expression and activating downstream AKT signaling. Through a c-Met/STAT3/p300 pathway, lnc-TALC recruits the histone acetyltransferase p300 to the MGMT promoter, increasing H3K9/27/36 acetylation and elevating MGMT levels. The result is enhanced O^6-methylguanine repair and robust TMZ resistance in GB cells [87]. Additionally, lnc-TALC may alter the GB microenvironment and decrease tumor sensitivity to TMZ chemotherapy. This suggests that the cross-talk between GB cells and microglia mediated by lnc-TALC may inhibit the effectiveness of chemotherapy and suggest possible combination therapy approaches to address TMZ resistance in GB [88].

***H19******:*** The imprinted lncRNA H19 is also implicated in TMZ resistance. H19 is overexpressed in resistant GB and acts as a decoy for miR-138-5p and miR-22-3p. Both miRNAs normally target BMP2 mRNA. By sponging miR-138/miR-22, H19 de-represses BMP2 expression. BMP2 signaling then promotes GB cell survival under TMZ. Thus, H19 enhances chemoresistance via a miR-138/22–BMP2 regulatory axis [89,90].

***PVT1:*** The lncRNA PVT1 is highly expressed in gliomas and correlates with aggressive progression. PVT1 drives TMZ resistance by engaging the JAK/STAT pathway. Transcriptomic analyses show PVT1 positively correlates with IL6, JAK3, STAT1/3, etc., and PVT1 knockdown significantly reduces JAK3 and STAT3 protein levels. By activating IL6/JAK/STAT signaling, PVT1 promotes cell survival and therapy resistance. In GB models, PVT1 depletion enhances TMZ sensitivity, confirming its role as a chemoresistance mediator [[91], [92], [93]].

#### 4.2.1 LncRNAs exert their regulatory functions through several mechanisms

***Tumor Growth:*** By interacting with chromatin-modifying complexes, lncRNAs like HOTAIR can enhance the expression of oncogenes, driving tumor growth. They can also recruit transcription factors to specific gene loci, influencing the transcriptional landscape of GB cells.

***Stem Cell Maintenance:*** Certain lncRNAs are involved in maintaining cancer stem cell populations, which contribute to tumor heterogeneity and the capacity for self-renewal. This characteristic is crucial for the resilience of GB against therapeutic interventions.

**Fig. 4:**
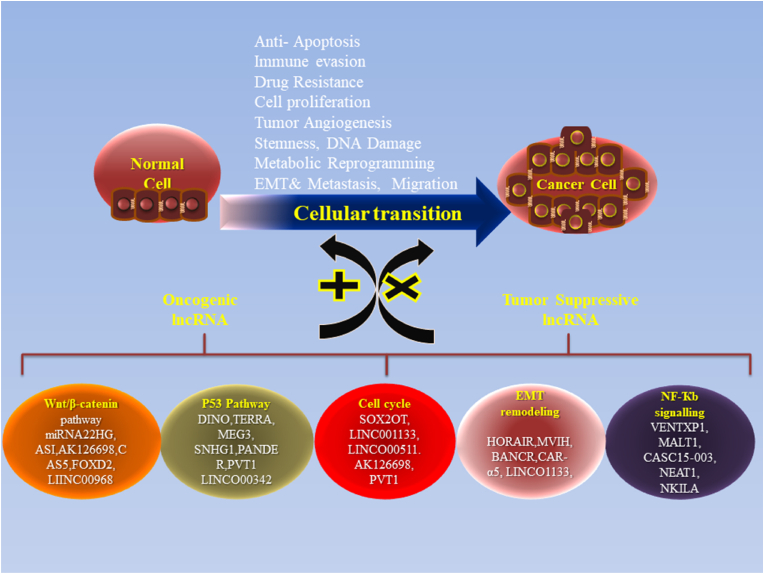
Functional Roles of oncogenic and tumor-suppressive lncRNAs in cancer: Oncogenic lncRNAs promote tumorigenesis by enhancing cell proliferation, inhibiting apoptosis, and facilitating metastasis, while tumor-suppressive lncRNAs counteract these processes to prevent cancer progression.

***Immune Modulation:*** LncRNAs can influence the immune landscape within the TME. By regulating the expression of cytokines and immune checkpoint molecules, lncRNAs may affect the recruitment and activity of immune cells, allowing GB to evade immune surveillance [94] Fig. 4.

### 4.3 Circular RNAs (circRNAs) and their emerging role in GB

These are unique, covalently closed RNA molecules formed by back-splicing of exons. CircRNAs are often stable and resistant to degradation, allowing them to serve as important regulators in the cell. They can function as sponges for miRNAs, binding to them and preventing their interaction with target mRNAs, thus modulating gene expression [95,96]. The study of ncRNAs has revealed their critical involvement in the pathogenesis of various cancers, including GB, where they play key roles in tumor growth, metastasis, and therapeutic resistance. CircRNAs are a novel class of ncRNAs that have garnered attention for their unique structures and regulatory roles in GB.

Key examples of circRNAs include

***circ-FBXW7:*** This CircRNA acts as a sponge for miR-197, thus enhancing the expression of FBXW7, a tumor suppressor that plays a vital role in regulating cell proliferation and survival by down-regulating miR-197 [97,98]. circ-FBXW7 promotes the degradation of oncogenic proteins, thereby inhibiting GB progression.

***circHIPK3:*** Another important circRNA, circHIPK3, is involved in regulating cell proliferation and apoptosis in GB. It sponges several miRNAs, influencing the expression of genes that control these critical cellular processes [99]. CircHIPK3 is an endogenous RNA that competes with other RNAs and primarily uses miRNA sponging to cause cancer. miR-654 is one of its best-studied targets; it typically suppresses tumors by preventing the expression of genes linked to metastasis and proliferation. CircHIPK3 absorbs miR-654 to alleviate the inhibition of IGF2BP3, a protein that promotes tumor growth and stabilizes oncogenic transcripts [100]. CircHIPK3 has also been shown to sponge miR-124, a well-known brain-specific tumor suppressor miRNA, which promotes the migration and proliferation of glioma cells by derepressing its downstream targets, including CDK6 and STAT3 [101]. The significance of circHIPK3 in regulating miRNA activity and influencing the pathophysiology of gliomas is demonstrated by these interactions.

CircRNAs can modify interactions between proteins and between proteins and RNA by serving as scaffolds or decoys for RNA-binding proteins (RBPs). Circ-FBXW7, for example, has been demonstrated to interact with and stabilize the tumor suppressor FBXW7. It can also be translated into a functional protein (FBXW7-185aa), which impedes the growth of GB by decreasing the stability of c-Myc [98]. Similar to this, circ-SHPRH encodes SHPRH-146aa, a tumor-suppressive protein that prevents full-length SHPRH from degrading and thereby stops the growth of gliomas [102]. CircRNAs are also involved in the regulation of transcription and alternative splicing. CircRNAs that are nuclear-localized, like circ-ITCH, work with the RNA polymerase II complex and U1 snRNP to enhance the transcription of their parent genes [103]. Furthermore, through intricate networks of RNA–protein and RNA–RNA interactions, circRNAs can contribute to stemness, invasion, and resistance to treatment in GSCs. The potential of circRNAs as GB therapeutic targets and diagnostic markers is highlighted by their diverse roles.

#### 4.3.1 Mechanism of regulatory role of circRNAs in sponging miRNA

CircRNAs primarily function as miRNA sponges, sequestering miRNAs and preventing them from binding to their target mRNAs. This sponging activity can lead to the upregulation of oncogenes or downregulation of tumor suppressors, thereby impacting GB biology. By modulating miRNA availability, circRNAs can significantly influence gene expression and contribute to the aggressive nature of GB.

### 4.4 Enhancer RNA (eRNA)

Enhancer RNAs (eRNAs) are ncRNAs transcribed from enhancer regions, and their expression abundance reflects the activity of enhancers [104]. They are typically short, non-polyadenylated, and nuclear-localized, playing roles in promoting chromatin accessibility and facilitating transcriptional activation of target genes. Therefore, elucidating the mechanism of eRNA regulation in gliomas is likely to provide valuable insights into the pathogenesis of both primary and recurrent gliomas. eRNAs might contribute to enhancer activity and facilitate the formation of enhancer-promoter loops through the recruitment of RNA polymerase II and various TFs, thereby modulating the transcription of target genes [105,106].

Recent studies have demonstrated that enhancers can maintain a drug-resistant state via their targeted transcriptional programs [107,108]. Therefore, identifying drug response–related eRNAs and their regulatory programs might contribute to the development of precision therapies and biomarkers for gliomas. The global dynamic expression landscape of eRNAs during the initiation and progression of primary and recurrent gliomas, including LGG and GB, reveals that most eRNAs are highly dynamically expressed in different stages of gliomas, suggesting that eRNAs might have stage-specific characteristics [108,109].

eRNAs like TMZR1-eRNA, derived from the STAT3 locus, have been found to regulate key signaling pathways in GB. These eRNAs can affect the expression of oncogenes, thereby affecting tumor growth and survival. Certain eRNAs regulate GB cell sensitivity to TMZ, a standard chemotherapeutic agent. Specifically, TMZR1-eRNA inhibition has been shown to decrease the expression of STAT3, a protein linked to chemotherapy resistance, which has been shown to enhance the efficacy of treatment in GB cells [110]. These studies suggest that these molecules could serve as potential biomarkers for prognosis and therapeutic targets in GB treatment. The identification of eRNAs specific to glioma stem cells raises the possibility of designing tailored RNA-based therapies aimed at these resistant cell populations. In a study using chromatin immunoprecipitation sequencing (ChIP-seq), researchers found that GB stem cells contained multiple eRNAs that were specific to GB cells. Significant correlations were found between certain eRNAs and patient outcomes, emphasizing the potential for eRNAs to act as novel epigenetic regulators within tumorigenesis [108].

### 4.5 Exosomal ncRNAs in GB

Exosomal ncRNAs play a crucial role in regulating GB pathways, and influencing tumor progression and therapeutic responses. For instance, miR-21 is often upregulated in GB, promoting cell proliferation by targeting tumor suppressor genes like PTEN, which enhances survival and growth. The 3′ untranslated region (3′-UTR) of PTEN mRNA is directly bound by miR-21, which results in post-transcriptional repression. By constitutively activating this prosurvival signaling cascade, the ensuing downregulation of PTEN, a tumor suppressor and negative regulator of the PI3K/AKT pathway, promotes glioma cell invasion, proliferation, and resistance to apoptosis [111,112]. Similarly, miR-221/222 inhibits pro-apoptotic factors, further aiding cell survival, while exosomal lncRNAs such as H19 can drive cell cycle progression [113,114]. In terms of invasion, miR-10b enhances the migratory capacity of GB cells by targeting genes involved in cell adhesion, and exosomes can promote epithelial-mesenchymal transition (EMT), facilitating local invasion. Exosomal ncRNAs also contribute to angiogenesis, with miR-125b promoting blood vessel formation, and they play a role in immune evasion by modulating immune responses through miR-155, which helps the tumor escape immune detection [70,114]. Additionally, ncRNAs are implicated in therapeutic resistance; for example, downregulation of miR-34a can lead to increased resistance to chemotherapy. Finally, ncRNAs can enhance cancer stem cell properties, contributing to tumor maintenance and recurrence [70]. The complex interactions of exosomal ncRNAs in these pathways highlight their potential as targets for therapeutic intervention and as biomarkers for GB management.

### 4.6 NcRNAs in therapeutic resistance

The role of ncRNAs in therapeutic resistance is a critical area of research in GB. ncRNAs influence resistance mechanisms through various pathways:

***Chemotherapy Resistance:*** NcRNAs can regulate the expression of genes involved in drug metabolism, efflux, and apoptosis. For instance, certain lncRNAs can enhance the expression of drug transporters, leading to decreased efficacy of chemotherapeutic agents. ABCB1 (P-glycoprotein), a well-characterized ABC transporter that is known to efflux chemotherapeutic agents from tumor cells, is upregulated by the lncRNA SBF2-AS1, promoting TMZ resistance [85]. Multidrug resistance-associated protein 1 (MRP1/ABCC1), another efflux pump involved in drug clearance, has also been demonstrated to be activated by lncRNA H19, increasing resistance [115]. Additionally, miRNAs may target pro-apoptotic genes, promoting cell survival and resistance to chemotherapy [65,70,73].

***Radiotherapy Resistance:*** NcRNAs are implicated in the cellular response to radiation therapy. Some miRNAs can enhance DNA repair pathways, allowing GB cells to survive radiation exposure and continue proliferating [73].

***Targeted Therapy Resistance:*** NcRNAs can also influence the expression of targets for specific therapies. For example, lncRNAs may modulate the expression of receptor tyrosine kinases, affecting the sensitivity of GB to targeted therapies [114].

## 5 Interplay between progenitor cells, microglia, and NcRNAs in GB

### 5.1 Progenitor cell-microglia cross-talk

The interaction between progenitor cells and microglia within the GB-TME is a crucial factor driving tumor progression [116]. GSCs, which originate from transformed neural progenitor cells, actively influence microglial behavior through direct and indirect signaling mechanisms [117]. Roles of progenitor cells, microglia, and ncRNAs in pathogenesis and therapeutic resistance in GB are summarized in Table 1.

**Table 1:** Roles of progenitor cells, microglia, and ncRNAs in GB pathogenesis and resistance.

Types	Role in GB Pathogenesis	Mechanism of Resistance	Ref
**Progenitor Cells**			
Neural Progenitor Cells (NPCs)	Provide cells with self-renewal and differentiation potential; Mutations can trigger tumorigenic transformation.	High drug-efflux pump activity, enhanced DNA repair, and maintenance of stemness properties.	[131]
GSCs	Promote tumor growth and recurrence with stem-like properties and contribute to GB heterogeneity.	Quiescence, increased DNA repair, hypoxic niche protection	[132]
Oligodendrocyte Progenitor Cells (OPCs)	Potential cell of origin in the proneural GB; Dysregulation of OPCs promotes tumor progression	Activation of PI3K/Akt/mTOR signaling pathways; Adaptation to microenvironmental stressors	[133,134]
Mesenchymal Progenitor Cells (MPCs)	Differentiation into tumor-associated stromal cells; supports aggressive growth of the mesenchymal subtype.	Enhance invasion, angiogenesis, and immune evasion	[135]
Endothelial Progenitor Cells (EPCs)	Support neovascularization, increase blood supply to the tumor, and facilitate invasion.	Maintain a hypoxic environment, protect from radiotherapy, and support angiogenesis.	[136]
**Microglia**			
Tumor-Associated Microglia/Macrophages (TAMs)	Support tumor growth through secretion of growth factors and cytokines; promote GB invasion and vascularization.	Immunosuppressive environment, increased secretion of anti-inflammatory cytokines	[137]
M1 Microglia (Pro-inflammatory)	Transiently suppress GB progression by releasing pro-inflammatory cytokines (e.g., TNF-α, IL-1β)	Reduced activity due to tumor-derived immunosuppressive signaling and metabolic reprogramming	[138]
M2 Microglia (Anti-inflammatory)	Promote tumor growth by enhancing angiogenesis, immunosuppression, and extracellular matrix remodeling.	High resistance through secretion of growth factors (e.g., TGF-β) and anti-inflammatory cytokines	[35,139]
Reactive Microglia	Activated in response to GB-induced inflammation; secretes factors promoting GB proliferation and matrix remodeling.	Secrete MMPs that support tumor invasion	[140]
Perivascular Microglia	Facilitate the invasion of GB cells along blood vessels and contribute to the formation of the perivascular niche.	Protect tumor cells by promoting a supportive niche and maintaining BBB integrity.	[141]
Glioma-Associated Microglia (GAMs)	Specialized microglia in GB interact closely with GSCs and tumor cells to promote proliferation and invasion.	Promote therapeutic resistance by maintaining stemness and supporting immune evasion.	[142]
**Non-Coding RNAs**			
miR-21	Promotes GB cell proliferation and invasion and inhibits apoptosis by targeting tumor suppressor genes (e.g., PTEN, PDCD4).	Increases resistance by activating anti-apoptotic signaling pathways and reducing sensitivity to chemotherapy	[143,144]
miR-10b	Facilitates tumor cell invasion and promotes stem cell-like properties	Induces therapeutic resistance through upregulation of pro-survival pathways and inhibition of apoptosis	[145]
HOTAIR	Enhances GB cell migration, invasion, and EMT	Contributes to radioresistance by promoting DNA damage repair and enhancing stemness properties	[77,146,147]
MALAT1	Supports tumor growth and angiogenesis through modulation of gene expression	Enhances resistance by modulating autophagy and promoting anti-apoptotic mechanisms	[79,148,149]
circHIPK3	Promotes GB proliferation and invasiveness by sponging tumor-suppressive miRNAs (e.g., miR-124)	Mediates chemoresistance through PI3K/AKT signaling activation	[99,150]
SNHG12 (Small Nucleolar RNA Host Gene 12)	Enhances GB proliferation, migration, and immune evasion	Increases resistance by modulating immune checkpoints and enhancing anti-apoptotic signaling	[151,152]
miR-155	Promotes tumor progression by targeting tumor suppressor genes and facilitating immunosuppression	Contributes to radioresistance and chemoresistance by improving DNA repair mechanisms	[153,154]

***Progenitor Cell Influence on Microglia******:*** Progenitor cells release various signaling molecules, including chemokines and cytokines, which affect microglial polarization [118]. This polarization shifts microglia towards a tumor-supportive phenotype, often described as M2-like polarization [35]. These M2-polarized microglia support the immunosuppressive and pro-tumorigenic environment by releasing factors that promote glioma growth and inhibit anti-tumor immune responses [35,37].

***Microglial******Support******for GSCs******:*** Microglia, in response to progenitor cell signals, secrete various cytokines (IL-6, TGF-β) and growth factors (CSF-1, VEGF) that enhance the survival, self-renewal, and proliferation of GSCs [34,37]. This reciprocal interaction between progenitor cells and microglia fosters a symbiotic relationship where both cell types promote each other's survival, facilitating tumor growth, invasion, and resistance to therapies [37,38,118].

### 5.2 ncRNAs as mediators of cellular interactions

NcRNAs, which include miRNAs, lncRNAs, and circRNAs, have emerged as key regulators of cellular communication within the GB-TME [72]. They modulate the cross-talk between progenitor cells and microglia, influencing the course of GB development [72,73].

Subtype-specific miRNA expression profiles have been demonstrated in recent research to be important in promoting or sustaining these transcriptional states. For example, miR-10b and miR-21, which are linked to invasion and proliferation, are enriched in the mesenchymal subtype and support its aggressive characteristics [119]. On the other hand, miR-128, miR-34a, and miR-137 are primarily expressed in the proneural subtype, where they target genes linked to stemness to control differentiation and inhibit carcinogenesis [120]. In addition to being potential biomarkers for diagnosis or prognosis, these miRNA signatures provide information about treatment vulnerabilities specific to each subtype of GB.

***ncRNA-Mediated Modulation of Communication******:*** ncRNAs can act as molecular bridges, modulating signaling pathways and transcriptional networks between progenitor cells and microglia [74,121]. For instance, miRNAs such as miR-124 and miR-21 are known to regulate microglial polarization and progenitor cell behavior, either suppressing anti-tumor responses or promoting the M2-like phenotype that supports tumor growth [61,62,121].

***Regulatory Feedback Loops******:*** NcRNAs can establish complex regulatory feedback loops. For example, miRNAs may inhibit the expression of specific transcription factors that would otherwise limit progenitor cell proliferation, while lncRNAs and circRNAs may act as “sponges” for these miRNAs, reducing their activity and thus maintaining the stem-like state of glioma cells. These regulatory interactions create a finely tuned system that promotes GB progression [62,95,113,122].

***Impact on TME******:*** NcRNAs not only affect individual cells but also modulate the broader TME [123]. They influence the secretion of cytokines and growth factors, reshape immune cell recruitment, and alter the extracellular matrix composition, thereby facilitating tumor-promoting conditions. The dysregulation of ncRNAs amplifies cellular cross-talk, reinforcing GB malignancy [61,73,113].

### 5.3 NcRNAs-microglia in GB progression

**miR-155:** MiR-155, a well-known pro-inflammatory miRNA, is upregulated in M1-type activated microglia. Because it inhibits tumor-supportive pathways and increases the production of inflammatory cytokines, it enhances anti-tumor responses in the GB microenvironment. But neurotoxicity can also result from over-activation [124].

**miR-124:** A quiescent, anti-inflammatory state is maintained by miR-124, a miRNA that is abundant in resting (homeostatic) microglia. A change toward the tumor-supportive M2 phenotype is correlated with its downregulation in GB-associated microglia, which promotes the progression of GB [125].

**miR-146a:** In the NF-κB pathway, it targets IRAK1 and TRAF6 to function as a negative feedback regulator of inflammation. Upregulated miR-146a in microglia can inhibit pro-inflammatory reactions, which could lead to an immunosuppressive GB microenvironment [126].

**LncRNA GAS5:** GAS5 is known to be expressed in microglia and regulates phagocytic activity, cytokine production, and microglial apoptosis. Downregulation of GAS5 has been linked to reduced immune surveillance and tumor support [127].

### 5.4 Implications for tumor progression and resistance

The intricate interplay between progenitor cells, microglia, and ncRNAs creates a feedback system that accelerates GB progression and strengthens therapeutic resistance.

***Tumor Progression******:*** The continuous cross-talk between progenitor cells and microglia, mediated by ncRNAs, enhances the invasive capacity of GSCs, and promotes tumor heterogeneity [23,37,81]. This complex cellular and molecular environment supports the creation of a highly adaptive and aggressive tumor. GSCs, supported by microglial-derived factors and ncRNA signaling, maintain their self-renewal and invasive properties, contributing to the relentless growth of GB [5,15,19,20].

***Synergistic Roles in Therapeutic Resistance******:*** This tripartite interaction is also a major contributor to therapy resistance. Progenitor cells and GSCs exhibit high plasticity, which allows them to survive conventional treatments such as radiotherapy and chemotherapy [20,29,128,129]. Microglial-derived cytokines further protect these cells from therapy-induced apoptosis [37]. Moreover, ncRNAs can upregulate resistance-related genes, such as those involved in DNA repair and drug efflux, reinforcing the tumor's ability to withstand therapeutic pressure [60,122]. The synergistic action of these elements thus creates a robust, multi-layered defense against current treatment strategies [22,130].

## 6 Therapeutic implications and future directions

### 6.1 Current therapeutic strategies

GB treatment remains a significant challenge, particularly due to the involvement of progenitor cells, microglia, and ncRNAs in tumor progression and therapeutic resistance [130,155,156]. Current GB management includes surgical resection followed by adjuvant radiotherapy with TMZ, an alkylating agent (the most widely used chemotherapeutic drug for glioma management), and followed by chemotherapy alone [3,4,157,158]. Emerging therapeutic strategies target progenitor cells, microglia, and ncRNAs to disrupt the GB microenvironment and limit tumor growth [32].

**Treatments Targeting Progenitor Cells:** Therapeutic approaches aimed at progenitor cells and GSCs primarily focus on differentiation therapy and the inhibition of key signaling pathways [26,159,160]. Differentiation therapy attempts to drive GSCs into more differentiated, less tumorigenic states, thereby reducing their proliferative capacity [161,162]. Drugs that target critical signaling pathways, such as the Notch, Wnt, and Hedgehog (SHH) pathways, aim to inhibit the self-renewal and maintenance of stem cells [163,164].

**Microglia-Targeted Therapies:** Therapies targeting microglia seek to reprogram these immune cells from a pro-tumorigenic to an anti-tumorigenic state [50,165]. One promising approach involves using CSF-1R inhibitors to block signals that promote microglial support for GB growth. In addition, strategies to polarize microglia towards an M1-like phenotype (anti-tumor) or prevent their recruitment into the TME are being explored [35,48]. With better CNS penetration and durability than current treatments, ASOs show great promise as adjuvant therapy for high-grade gliomas. Even though early findings are promising, more investigation is required to confirm the efficacy and safety of ASO therapy in clinical settings. [166]. For example, researchers have developed ASOs designed to degrade the mRNA of the K27M variant, which is known to promote gliomagenesis. These ASOs have been shown to effectively reduce the levels of K27M mutant mRNA, thereby reversing aberrant epigenetic changes in preclinical models [167].

**ncRNA-Based Therapies:** The therapeutic potential of ncRNAs lies in their regulatory roles in gene expression and tumorigenesis [168,169]. Antisense oligonucleotides (ASOs), miRNA mimics, and miRNA inhibitors have been developed to target oncogenic ncRNAs or restore the function of tumor-suppressive ncRNAs [[170], [171], [172]]. For instance, miRNA mimics can be introduced to restore miRNA levels that suppress glioma growth, while inhibitors can block oncogenic miRNAs that contribute to tumorigenesis. Clinical trials are ongoing to assess the efficacy of ncRNA-based therapies in GB [170,[173], [174], [175], [176], [177]].

### 6.2 Challenges in targeting the progenitor cells-microglia-ncRNA axis

Despite advances in therapeutic approaches, targeting the progenitor cells-microglia-ncRNA axis presents several significant challenges:

**Blood-Brain Barrier (BBB):** The BBB is a major obstacle in delivering therapeutic agents to the brain. Its highly selective permeability limits the efficacy of many treatments, including small molecule inhibitors, antibodies, and nucleic acid-based therapies such as ASOs and miRNA mimics. Overcoming the BBB remains a critical hurdle in developing effective GB therapies [[178], [179], [180]].

**Tumor Heterogeneity:** GB is characterized by extreme tumor heterogeneity, not only in its genetic and epigenetic landscape but also in the behavior of GSCs and microglia [128,181]. This heterogeneity results in diverse treatment responses, with different tumor cell populations exhibiting varying levels of resistance [2]. Microglia and GSCs can adapt to therapeutic pressure, leading to recurrence even after aggressive treatment. These adaptive responses significantly complicate the development of effective therapies that can target all tumor subpopulations [5,20,37].

### 6.3 Emerging therapeutic approaches

To overcome these challenges, several emerging therapeutic approaches are being developed to address the multifaceted nature of GB pathogenesis.

***Combination Therapies******:*** Single-agent therapies have shown limited success due to the complex and adaptive nature of GB [55]. Combination therapies, which target multiple components of the TME simultaneously, are being explored to improve treatment outcomes [55,56,182]. For instance, co-targeting progenitor cell pathways (e.g., Notch or Wnt) along with microglia modulation (CSF1R inhibitors or immunomodulators) and ncRNA-based interventions could potentially address both the cellular and molecular components driving GB [48,163,183].

***Personalized Therapies Based on ncRNA Profiles******:*** The advent of precision medicine offers the possibility of tailoring treatments based on the specific ncRNA expression profiles of individual tumors [184]. Personalized therapeutic approaches could involve the use of miRNA mimics or inhibitors specifically chosen to target the dysregulated ncRNAs driving the patient's tumor [185]. This approach could help overcome tumor heterogeneity by targeting the unique molecular characteristics of each tumor [96,186,187].

***Advances in Drug Delivery Systems******:*** Recent advances in nanoparticle and exosome-based delivery systems show promise in enhancing drug delivery across the BBB and directly targeting glioma cells [179,188]. Nanoparticles can be engineered to carry therapeutic agents such as pathway inhibitors or ncRNAs, improving their bioavailability and specificity [189,190]. Exosomes, natural carriers of RNA and proteins, have emerged as a potential vehicle for delivering miRNA-based therapies to the tumor site, offering a novel approach to overcoming the BBB [[191], [192], [193], [194]].

## 7 Conclusion

GSCs, which arise from progenitor cells, are central to tumor initiation, recurrence, and resistance, due to their plasticity and self-renewal capabilities. The transformation of progenitor cells into GSCs, driven by genetic mutations and epigenetic changes, creates a pool of tumor-initiating cells that exhibit resistance to conventional therapies. Microglia, co-opted by GB cells, play a pivotal role in creating an immunosuppressive microenvironment that fosters tumor growth. The cross-talk between microglia and GSCs, mediated by cytokines and growth factors, enhances GSC survival and therapy resistance. Furthermore, ncRNAs, particularly miRNAs and lncRNAs, regulate the expression of key genes involved in tumor growth and the cellular interactions between progenitor cells and microglia. These ncRNAs also contribute to the maintenance of the stem-like phenotype in GSCs and modulate immune responses, further promoting tumor progression and resistance to treatments.

The complexity of GB, with its diverse and interconnected cellular and molecular components, necessitates the development of multi-targeted therapeutic approaches. Future research should focus on understanding the dynamic interactions between progenitor cells, microglia, and ncRNAs. Identifying the precise mechanisms by which these components communicate within the TME is crucial for designing more effective therapies. One promising avenue is the personalization of therapies based on individual tumor profiles, including ncRNA expression. Moreover, combination therapies that simultaneously target GSCs, microglia, and ncRNAs hold the potential for overcoming treatment resistance.

Understanding the roles of progenitor cells, microglia, and ncRNAs in GB pathogenesis opens novel therapeutic avenues that go beyond conventional treatments. Targeting the progenitor cells-microglia-ncRNA axis can potentially disrupt the cellular and molecular networks that drive tumor growth and therapy resistance. Novel therapies such as pathway inhibitors for progenitor cells, immunomodulatory agents targeting microglia, and ncRNA-based therapeutics are being developed to improve patient outcomes. These therapies, combined with advanced drug delivery technologies, could lead to more effective treatments that minimize resistance, slow tumor progression, and extend survival in patients with GB.

## CRediT authorship contribution statement

**Adil Husain:** Writing – review & editing, Writing – original draft, Visualization, Validation, Formal analysis, Conceptualization. **Firoz Ahmad:** Writing – review & editing, Writing – original draft, Investigation, Formal analysis, Conceptualization. **Sandeep Pandey:** Writing – original draft, Supervision, Project administration, Formal analysis. **Tarun Kumar Upadhyay:** Resources, Methodology, Formal analysis. **Sojin Kang:** Methodology, Funding acquisition. **Min Choi:** Funding acquisition. **Jinwon Choi:** Funding acquisition. **Moon Nyeo Park:** Funding acquisition. **Bonglee Kim:** Funding acquisition.

## Data availability statement

The article does not fall under the category of data sharing because no datasets were created or examined in this study.

## Funding

This research was supported by Basic Science Research Program through the 10.13039/501100003725National Research Foundation of Korea, 10.13039/100009950Ministry of Education (NRF-2020R1I1A2066868), the 10.13039/501100003725National Research Foundation of Korea (10.13039/100028114NRF), Korea Ggovernment (10.13039/501100014188MSIT) (No. 2020R1A5A2019413), a grant of the Korea 10.13039/100018696Health Technology R&D Project, 10.13039/501100003710Korea Health Industry Development Institute (10.13039/501100003710KHIDI), 10.13039/100009647Ministry of Health & Welfare, Republic of Korea (Grant Number: RS-2020-KH087790) and the 10.13039/501100003725National Research Foundation of Korea (10.13039/100028114NRF), Korea government (10.13039/501100014188MSIT) (RS-2024-00350362).

## Declaration of competing interest

The authors declare that they have no known competing financial interests or personal relationships that could have appeared to influence the work reported in this paper.
